# Assessing treatment response in thrombotic thrombocytopenic purpura: Beyond the platelet count

**DOI:** 10.1016/j.htct.2024.06.012

**Published:** 2024-11-07

**Authors:** Cilomar Martins de Oliveira Filho, Ibidunni Bode-Sojobi, Barbara D. Lam, Stephanie Conrad, Jonathan Berry, Brian J. Carney

**Affiliations:** a Mass General Brigham, Salem Hospital, Salem, Massachusetts, USA; b Beth Israel Deaconess Medical Center, Boston, USA

A 50-year-old male with systemic lupus erythematous presented with fever, abdominal pain, and diarrhea. Hemoglobin was 10 g/dL, near patient's baseline, and there was new thrombocytopenia with a platelet count 109 × 10^9^/L. The following day, platelets dropped to 24 × 10^9^/L. Hemolysis parameters were unremarkable. Treatment for immune thrombocytopenic purpura with IV dexamethasone was started but there was no improvement in the platelet count. On hospital Day 7, he developed a seizure, and was intubated. He evolved with shock and renal failure requiring dialysis. Lactate dehydrogenase rose to 1054 U/L and haptoglobin became undetectable. A peripheral blood smear revealed a large population of schistocytes. The PLASMIC score was 6.^1^ ADAMTS13 assay was done, the patient received fresh frozen plasma (FFP) and was transferred to a tertiary center for daily plasmapheresis with full FFP replacement. The platelet counts initially rose, then remained at around 50 × 10^9^/L on subsequent days. Notably, hemolysis parameters rapidly normalized. The population of schistocytes steadily decreased. Antiphospholipid antibodies and enterohemorrhagic *E. coli* tests were negative. On Day 5 of plasmapheresis, ADAMTS13 activity was undetectable, confirming a diagnosis of acquired thrombotic thrombocytopenic purpura. By Day 11 on plasmapheresis the patient improved consistently and was extubated (Figure 1).

**Figure 1:**
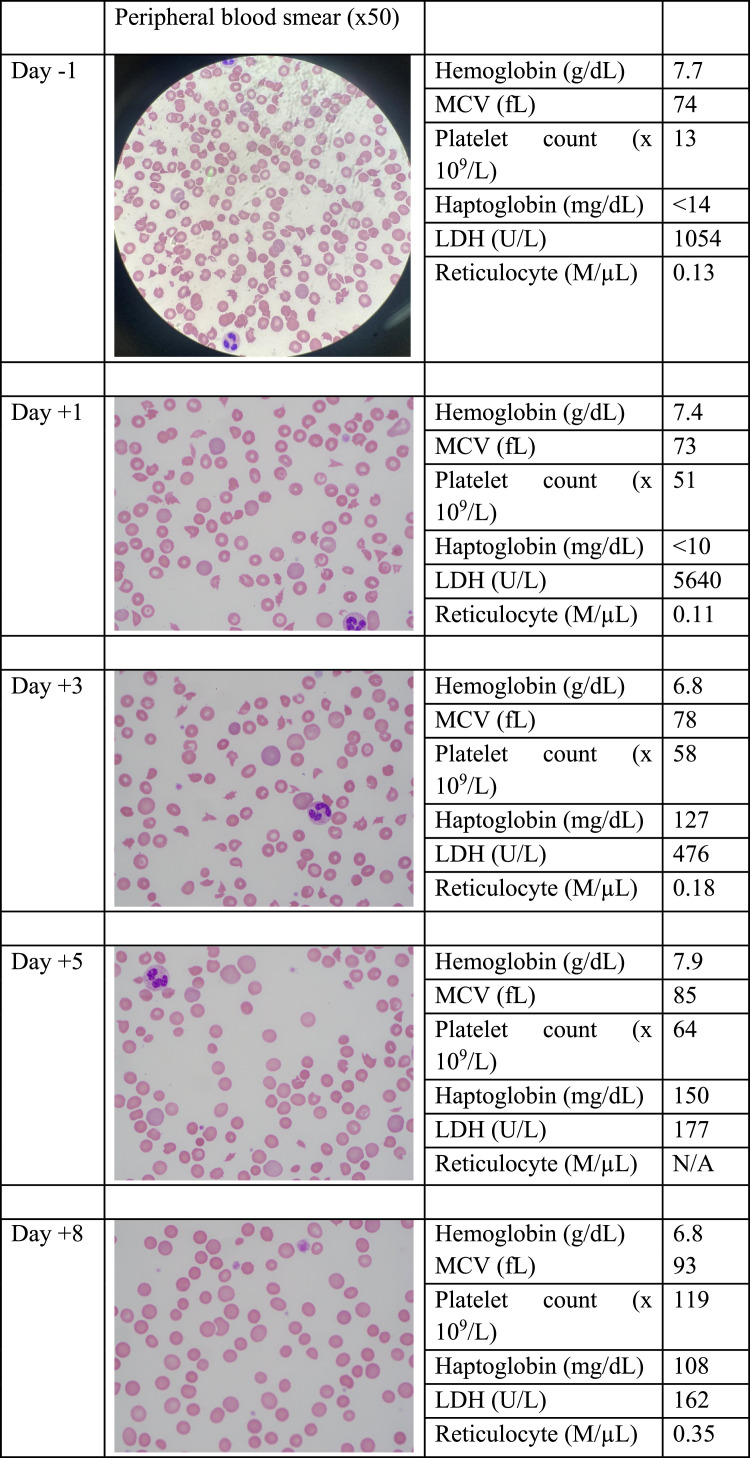
Days on plasmapheresis and changes in blood smears (x50), blood counts, and hemolysis parameters.

## Conflicts of interest

None.
